# Overdose deaths before and during the COVID-19 pandemic in a US county

**DOI:** 10.3389/fpubh.2024.1366161

**Published:** 2024-05-27

**Authors:** C. Hendricks Brown, Kimberly A. Johnson, Holly A. Hills, Wouter Vermeer, Dianne L. Clarke, Joshua T. Barnett, Reta T. Newman, Tim L. Burns, William A. Pellan

**Affiliations:** ^1^Department of Psychiatry and Behavioral Sciences, Feinberg School of Medicine, Northwestern University, Chicago, IL, United States; ^2^Department of Mental Health Law and Policy (MHC 2636), College of Behavioral and Community Sciences, University of South Florida, Tampa, FL, United States; ^3^Operation PAR, Inc., Pinellas Park, FL, United States; ^4^Department of Human Services, Pinellas County Government, Clearwater, FL, United States; ^5^Pinellas County Forensic Lab, District Six Medical Examiner Office, Largo, FL, United States; ^6^District Six Medical Examiner Office, Largo, FL, United States

**Keywords:** data driven decision support, opioid treatment, naloxone, excess non-COVID overdose deaths, drug seizures, drug arrests, data dashboards, harm reduction

## Abstract

**Introduction:**

Globally, overdose deaths increased near the beginning of the COVID-19 pandemic, which created availability and access barriers to addiction and social services. Especially in times of a crisis like a pandemic, local exposures, service availability and access, and system responses have major influence on people who use drugs. For policy makers to be effective, an understanding at the local level is needed.

**Methods:**

This retrospective epidemiologic study from 2019 through 2021 compares immediate and 20-months changes in overdose deaths from the pandemic start to 16 months before its arrival in Pinellas County, FL We examine toxicologic death records of 1,701 overdoses to identify relations with interdiction, and service delivery.

**Results:**

There was an immediate 49% increase (95% CI 23–82%, *p* < 0.0001) in overdose deaths in the first month following the first COVID deaths. Immediate increases were found for deaths involving alcohol (171%), heroin (108%), fentanyl (78%), amphetamines (55%), and cocaine (45%). Overdose deaths remained 27% higher (CI 4–55%, *p* = 0.015) than before the pandemic through 2021.Abrupt service reductions occurred when the pandemic began: in-clinic methadone treatment dropped by two-thirds, counseling by 38%, opioid seizures by 29%, and drug arrests by 56%. Emergency transport for overdose and naloxone distributions increased at the pandemic onset (12%, 93%, respectively) and remained higher through 2021 (15%, 377%,). Regression results indicate that lower drug seizures predicted higher overdoses, and increased 911 transports predicted higher overdoses. The proportion of excess overdose deaths to excess non-COVID deaths after the pandemic relative to the year before was 0.28 in Pinellas County, larger than 75% of other US counties.

**Conclusions:**

Service and interdiction interruptions likely contributed to overdose death increases during the pandemic. Relaxing restrictions on medical treatment for opioid addiction and public health interventions could have immediate and long-lasting effects when a major disruption, such as a pandemic, occurs. County level data dashboards comprised of overdose toxicology, and interdiction and service data, can help explain changes in overdose deaths. As a next step in predicting which policies and practices will best reduce local overdoses, we propose using simulation modeling with agent-based models to examine complex interacting systems.

## Introduction

The toll on humans across the globe due to COVID-19 is truly devastating. Globally, the number of deaths due to SARS COVID-2 infection was unprecedented among all infectious diseases since the 1918 H1N1 Pandemic, with between 2.5 and 5.4 million deaths across the world, and causes from non-COVID deaths were even higher, contributing upwards of 15 million excess deaths worldwide (1, 2). In the United States, there were over 1 million deaths due to COVID recorded to date (3, 4). Among risk groups in the United States, those who have substance use disorders are more likely to die from COVID (5). particularly among Black, non-Hispanic male persons (6). However, a large portion of excess deaths in the U.S. were due to overdose; compared to 71,000 deaths in 2019 before the pandemic, overdose deaths exceeded 91,000 in 2020 and 106,000 deaths in 2021 (7). In this paper we conduct detailed analyses of toxicologic, interdiction, and service system data that focus on some potential explanations of increasing overdose deaths during the pandemic within one county that, as we describe below, had an elevated level of excess overdose deaths during the pandemic compared to other U.S. counties.

Our focus on one county's experience in this paper deserves an explanation. It is true that federal, state, as well as local policies and practices regarding opioid and other substance prevention, treatment, rescue, and recovery all changed at varying times through the pandemic (8). Thus, a wide range of contexts can be used to examine overdose deaths before and during COVID.

**Figure 1:**
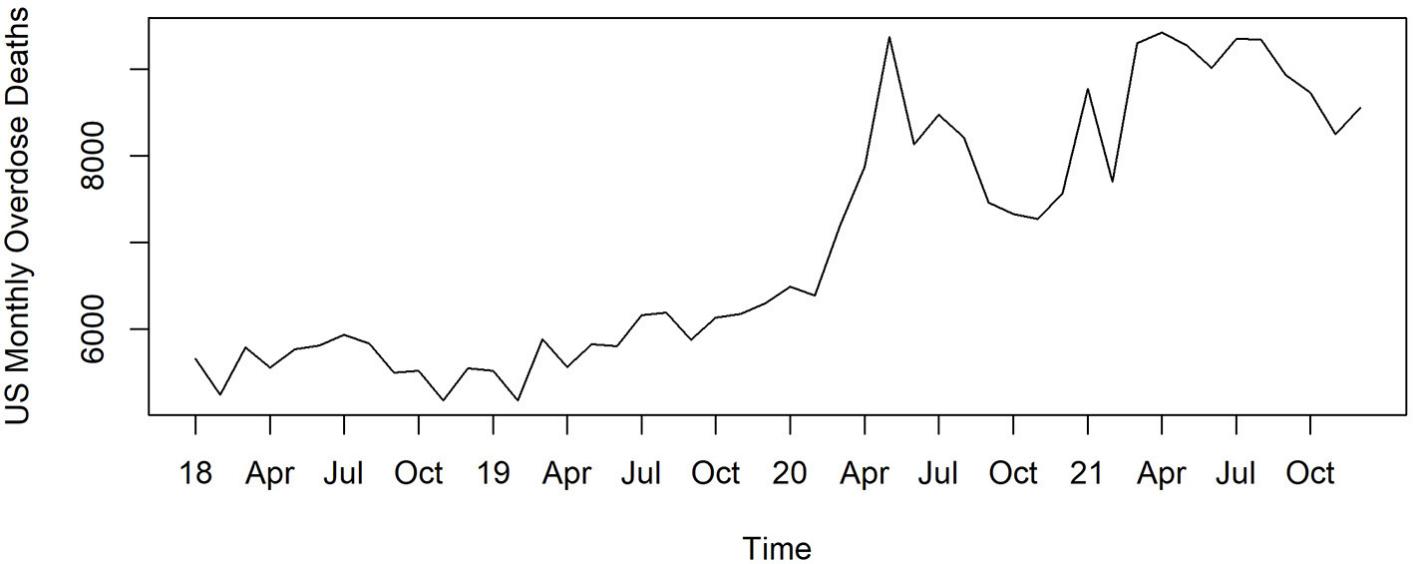
Overdose deaths by month for the United States 2018–2021.

In the U.S., the Centers for Disease Control and Prevention (CDC) reported a 28% increase in overdose deaths in the US starting in April 2020 when COVID deaths in the US first exceeded 1,000 per day (9). Monthly changes show this elevation started before the pandemic hit, then dropped 20% before increasing again through 2021 (Figure 1). While there was a loosening of national drug policies for take-home methadone in response to the pandemic crisis, there are challenges in concluding causal interpretations while tracking national overdoses over time (8).

In countries such as the United States that do not have a standard national drug prevention and treatment system, policies and practices that matter to the lives of people who use drugs are implemented mostly at the local level, usually a county or city. For example, some counties provide extremely limited access to and insurance coverage for methadone treatment, and some are averse to harm reduction approaches. This is a major reason that this paper takes a local approach of mapping a county's experience of overdose deaths before, at the start, and during the COVID pandemic. A second reason is that counties have experienced not only the pandemic with varying intensity and timing, but also varying causes of overdose deaths and which members of their society are most affected. For example, in Los Angeles County CA, evaluators found increased rates of opioid overdose during the pandemic for males, middle-aged and older adults (10) whereas in Santa Clara County, CA, the overdose rates were highest among 20 to 24 year olds (11).

Besides these local differences in COVID-19 and overdose exposures, all counties and municipalities were obliged to reconfigure service offerings in response to capacity restrictions imposed by the pandemic. We hypothesize that users' risk of overdose is affected not only by the available drug market and drug mixtures they take, but also by their exposure to interdiction, addiction, and social services, all of which are under the auspices of the county in most states. In particular, we hypothesize that drug seizures and drug arrests, access to resources for rescue, linkage to treatment, harm reduction, and recovery all influence overdose deaths. The natural experiment caused by the county level policy response to the pandemic can provide insight into the impacts of policy changes during and beyond the acute phase of the pandemic. Thus, a detailed mapping of county-level changes in decedent toxicologic findings and interdiction and services before, at the beginning and through the pandemic provides a deeper, locally relevant understanding of these relationships with overdoses that can better inform local decision making. This paper focuses on the overdose experience of Pinellas County before, at the start of the pandemic to the end of 2021.

Pinellas County has seen a significant growth in overdose deaths, as the annual overdose rate based on reporting of all accident, intentional, or undetermined deaths from psychoactive drugs has nearly doubled from 29 to 53 per 100,000 in the period from 2016 to 2020; the latter (age-adjusted) rate is higher than 81% of all US counties in the same year of 2020 (12). We estimate there are ~18,000 (3% of adults) persons who have opioid use disorder in Pinellas County.

At the start of the pandemic, curtailment policies changed rapidly in Florida. On March 17, 2020, the Governor ordered the state's bars, restaurants, and public schools to be closed for 30 days. On April 29, 2020, the Governor issued orders to determine conditions under which Florida's beaches, businesses, and institutions for all counties would reopen through the SafeSmartStepbyStep Plan (13). While Pinellas beaches were formally opened on May 4, 2020, numerous people were on the beaches while formally closed. All Pinellas County businesses except bars and nightclubs were allowed to reopen by June 5, 2020, and a clearance of full capacity for all restaurants and bars was declared by the Governor on September 25, 2020.

### Overview of Pinellas County's substance use services prior, at the beginning and during the pandemic

Before the pandemic, public treatment settings provided Medication for Opioid Use Disorder (MOUD) to over 2,000 persons daily among an estimated 18,000 with opioid use disorder. Regarding the county's non-profit opioid treatment services, drug screens were suspended March 18, 2020, residential admissions stopped for all but pregnant women on March 23, and admissions to methadone medication stopped 2 days later, as induction on methadone required face-to-face examination, something impossible without sufficient personal protective equipment that was not available to addiction treatment programs at the start of the pandemic. For stable patients, “take-home” medications rules were relaxed in Florida under an emergency order from the Substance Abuse and Mental Health Services Administration (SAMHSA) (14). Remote services offering buprenorphine induction started March 30. During the pandemic when x-waivers were still required, there were 120 listed buprenorphine prescribers, with only 5 licensed to care for up to 100 patients. By May 11, 2020, residential admissions reopened, and new patients could receive methadone medication.

Prior to the pandemic, there was negligible use of financial contingency management, the only approved treatment for stimulants, because of federal and state restrictions on using financial incentives for individuals who use drugs. It was only after this paper's study period (2019–2021) that federal funding was changed to allow $75 for contingency management (15). Florida prohibited state funding of financial incentives, so incentives are primarily limited to take-home methadone privileges.

Involuntary screening and assessment of impairment due to substances (called Marchman screening) was stopped at the beginning of the pandemic with the governor's closure order. In the community, involuntary screening, and county jail screening both restarted on June 4, but in-jail screening was stopped again June 15 due to a COVID outbreak.

Drug treatment staff in the publicly funded OTP were all required to be tested for COVID in early August due to exposure to a positive case. This caused a reduction in treatment availability due to the frequency of positive tests for staff and subsequent mandatory quarantine. The only month in 2020 when there was no staff quarantined was September 2020, meaning there were repeated reductions and restarts in MOUD and other addiction service availability due to staffing issues related to the pandemic.

Since 2017 the county's Opioid Task Force has guided and coordinated community approaches to reduce overdose deaths (16). The task force continued to meet remotely throughout the pandemic. This paper is one outcome of a community-research partnership with the Opioid Task Force.

### Outline of the paper

We present this detailed examination of the immediate and longer-term influences of the pandemic on overdose death on one county, Pinellas County, Florida. While the findings we report relate to this single county, the analytical approach we describe in this paper for this one county would be appropriate for different counties that have data dashboards measuring overdose as well as variations by population and service provision over time. In addition to characterizing the drugs that are causal agents in overdose deaths through toxicologic findings, we examine the timing of changes that occur in delivery of treatment, police drug seizures, and rescue-related services, including emergency medical services (EMS) and naloxone distribution for opioid rescue. We also examine whether there is a differential impact of the pandemic by race, age, sex, and drugs involved (6). These data provide contexts that the county's decision makers can use to understand the impact of policies on local outcomes.

We also introduce an index of excess overdose deaths relative to excess non-COVID-19 deaths, which can compare an individual county's experience with that of other counties and the US.

Finally, we describe how the knowledge gained from toxicologic, sociodemographic, interdiction, and service data like that we use in this paper – a data driven decision approach – could use predictions from complex system science modeling to enhance local decision through predictions that can reflect community values, priorities, and resources.

## Methods

### Pinellas County population description

An estimated 972,000 persons live in Pinellas County; its population density makes it the most densely populated county in Florida (17) and in the 99th percentile among all US counties (18). While the county has a total of 280 square miles, it has 588 miles of coastline, which account for one of the ways that illegal drugs enter the county. Averaged over 2017 to 2021, 12% of the population lives below the poverty line, and 5% are unemployed. The population is primarily White persons (82%), 11% identify as Black persons, and 10% as Hispanic persons. Compared to White persons, poverty rates for Black persons are twice as high (22%) and 50% higher for Hispanics persons (15%).

### Data sources

We obtained data from the Medical Examiner and Forensic Lab on all deaths in the county having substance toxicity from 2019 to 2021. Only those whose deaths were classified as accidental, suicide, or undetermined overdose were included, thus excluding homicides, fetal deaths, and natural causes (e.g., dying of cancer with positive toxicology for illegal drugs). Deaths from individual drugs without psychoactive properties (e.g., acetaminophen, carbon monoxide) were excluded. Ninety-two percent of these 1,701 overdose deaths during these 3 years had toxicologic tests conducted by the medical examiner; the remaining 8% included toxicologic findings from a hospital. Drugs were classified into major groups, opioids, amphetamines, anxiolytics, antidepressants, inhalants, hallucinogens, alcohol, and opioid treatments (e.g., methadone). We identified which drugs were present in the toxicology report or assigned causality for death. It is often the case that more than one drug is determined to be causally linked to overdose death when multiple drugs are found. We report on drugs that were causally related to death.

Demographic data on decedents included age, race, and sex. As ethnicity was not recorded in the toxicology findings of overdoses, we cannot report findings by ethnicity.

### Analyses

We introduce an index to measure the percentage change in overdose deaths that occurred in the 12 months after the pandemic began relative to the 12 months before. This index, the ratio of excess overdose deaths relative to excess non-COVID-19 deaths (EOD), is defined as:


$$\begin{array}{l} \text{EOD}=\Delta \left(\text{OD}\right)/\left(\Delta \left(\text{D}\right)-\Delta \left(\text{CD}\right)\right) \end{array}$$

where Δ represents the change in that quantity after COVID vs. before over the same periods of time (here 12 months). EOD's numerator counts the number of excess overdose deaths (OD) while the denominator counts excess deaths from all causes (D) except COVID-19 (CD). We use this index to compare Pinellas County's excess overdose deaths relative to that of other counties in the U.S. In operationalizing this quantity, we obtained county level deaths by cause (ICD-10 cause of death code of U07.1 for COVID-19 and codes: X40–X44, X60–X64, X85, and Y10–Y14 for drug overdose, which excludes alcohol) from monthly tables provided by CDC's WONDER (19). These consistencies in definitions reduce double counting of individuals (e.g., dying from COVID-19 and overdose) and allow comparisons across counties and nationally. Higher values of EOD represent a larger ratio of excess overdose deaths relative to excess non-COVID deaths. EOD is typically between 0 and 1 but is negative if there are fewer non-COVID deaths over time. For interpretability, this index is non-defined if there is a negative change in overall deaths or if the denominator is negative.

We examined whether overdose death rates differed between three time periods: before, at the beginning of the pandemic, and since the pandemic through 2021. We considered April 2020 the start of the pandemic in Pinellas as this was the first month where COVID-19 deaths were reported.

We counted overdose deaths in each month from January 2019 through December 2021 and used diagnostic plots with smooth fits and a discontinuity in April 2020. We used quasi-Poisson modeling with a log-linear trend model before the pandemic and another trend afterwards, allowing for a discontinuity between the end of April and May 2020. We made this choice since there were zero COVID deaths during most days in April, especially in the first half of the month. Thus April was a transition month. To investigate sensitivity of this definition of the transition point, we reran the analyses treating April as the first “After COVID” month. For visualizing this transition month, our figures show a smooth plot up until the end of April 2020, along with a disconnected smooth plot beginning in May 2020. This quasi-Poisson modeling statistical model for repeated rare count data, such as monthly overdose numbers, adjusts for excess, unaccounted-for variation. We conducted four Wald-type tests that compare regression coefficients to their respective standard errors. Specifically, we examined (1) mean death rates before and after the pandemic began, (2) estimated change in the rate at the beginning of the pandemic comparing the fitted values' endpoints before and after the discontinuity, (3) the difference in slopes before and after the pandemic began, and (4) the difference in fitted rates at the end of 2021 to that just before the pandemic. Standard errors for these tests were obtained from the asymptotic variance-covariance estimates from quasi-Poisson modeling. The use of fitted values for estimating changes, vs. differences between actual observed rates (e.g., differences in monthly counts immediately before and after the pandemic) is generally a more conservative approach.

We also examined how changes in interdiction occurred before vs. after the pandemic by examining monthly numbers of drugs and types of drugs seized by police in the county. We report on numbers of overdose rescues by emergency medical services and police. These data were combined to identify unique occurrences. Analyses of changes in these data were similar to the analyses of overdose deaths described above. We conducted monthly counts of new patients and overall number of patients in medication for opioid use disorder (MOUD) from the single publicly funded provider in the county.

As a summary analysis, we examined how the 2019–2021 monthly overdose counts are related to monthly data from core measures of interdiction, treatment, and rescue that we considered time-sensitive factors, thus potentially related to monthly overdose rates. For interdiction we selected two measures, the county's drug arrests and police seizures of opioids, stimulants, and benzodiazepines, the three primary drug categories responsible for the vast majority of overdose deaths. For treatment, we chose the number of methadone treatments provided in the county's single public opioid treatment center (OTC), both in-clinic and take-home doses. We did not include other medications for opioid treatment, particularly buprenorphine, as the majority of those treated with these other medications were received outside OTCs and these data were not available. For rescue we selected the count of suspected overdose calls from police and emergency medical services. While the monthly distribution of Narcan kits supplied by the State to community organizations is known, timing of when these kits are actually used is not known. Time variables, including whether the month occurred after the beginning of the pandemic, linear trends over time, and their interaction with before and after the pandemic also served as predictors.

Our statistical analyses of monthly overdose deaths involved three steps: the first examining the strength of each of the covariates described above as individual predictors, the second an analysis using all these predictors plus interactions, and a third final set involving a backwards elimination procedure that includes only those terms that were significant at *p* = 0.10 or smaller. We analyzed the effects of potential predictors using concurrent monthly relationships as well as lagged models. We compute a time series generalized linear model using a Poisson likelihood with a common autoregressive structure [ARMA (1, 1)] and checked for sensitivity of the findings with a Negative Binomial model as well as models where predictors of monthly overdose were lagged by 1 month. We used glm() and tsglm() functions in the R Statistical Package version 4.2.2 (20)

#### Human subjects and ethics

IRB approval for secondary data analysis was obtained from the University of South Florida (IRB ID 001165). No individuals provide consent as this IRB provides a waiver of the requirement for consent. The IRB approval allows the study team to obtain private health information (PHI) from the following sources: Florida Department of Children and Families (DCF/IDS) – FASAMS, Pinellas County Criminal Justice Information System (CJIS), Pinellas County Human Services, Healthcare for the Homeless/FQHC EHR data, and Florida Department of Health EHR Data. Individual identifiers are stripped from analytic datasets. Separately, toxicologic information from decedents is considered non-human subjects research. Monthly death counts from CDC WONDER were also obtained for COVID and overdose; these are designated as non-human research. Counts with no identifiers for publicly available data from the Florida Health website are also considered non-human subjects research.

## Results

### COVID deaths in Pinellas County

April 2020 was the first month where any COVID deaths were reported in Pinellas County (*n* = 28). In 2020, 767 individuals in Pinellas County died from COVID, and its crude death rate was 76.5 per 100,000 (12). Total deaths from COVID-19, in Pinellas County, were 1,982 by the end of 2021 and had nearly doubled by the end of 2022 (21).

We present toxicological and demographic changes in overdose deaths, followed by interdiction, treatment services, and social services.

### Comparison of deaths caused by overdose prior to, beginning and during the pandemic

The EOD for Pinellas County for April 2020 to March 2021 compared to the previous 12 months was 0.28. This ratio exceeds the EOD for 75% of the counties with data available on CDC WONDER (21). The number of overdose deaths in the first year of the pandemic was more than 50% of those dying from COVID during the same time period.

**Figure 2:**
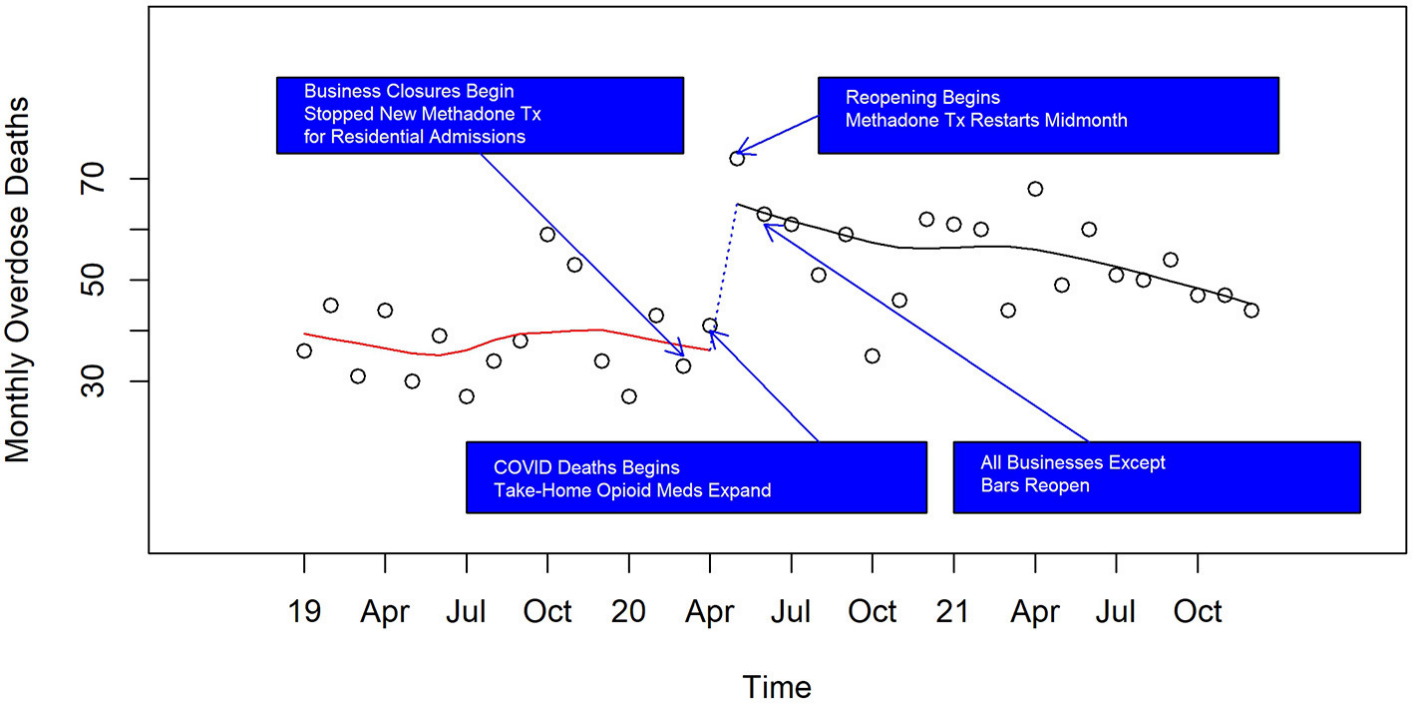
Monthly overdose deaths in Pinellas County before, at the beginning, and during the pandemic, 2019–2021.

Using our consistent definition of overdose deaths through the 3 years, there were 470, 596, and 635 respectively in the years 2019 to 2021, a total of 1,701 deaths. Averaging 47 overdose deaths per month over these 3 years, accidental overdoses comprise the largest number (44.5 per month) followed by suicide (2.1 per month) and undetermined (0.6 per month). From May 2020, until the end of 2021, the number dying from overdose is 40% higher than the previous period from January 2019 to April 2020 (95% confidence interval an increase of 27% to 55%, *p* < 0.00001). We then examined whether there was an initial increase in overdose deaths around April 2020. Figure 2 demonstrates a consistent rate of 40 overdoses per month before the pandemic (red line) followed by an abrupt 50% increase to above 60 per month (dashed line) with a gradual decline to below 50 per month, still elevated as compared to before the pandemic (black line). The highest count of monthly overdose deaths was in May 2020 with over 70 deaths.

On the first of these plots (Figure 2) displaying counts of monthly overdose death, we marked four state-wide events of rapid opening and closing of services due to COVID. As indicated above, on March 17, 2020, the Governor ordered the state's bars, restaurants, and public schools to be closed for 30 days. Then at the end of April 2020, the Governor issued orders to determine conditions under which Florida's beaches, businesses, and institutions for all counties would reopen through the SafeSmartStepbyStep Plan (13). Pinellas beaches were formally opened in early May, 2020, but many people were on the beaches while formally closed. All Pinellas County businesses except bars and nightclubs were allowed to reopen by early June, 2020, and a clearance of full capacity for all restaurants and bars was declared by the Governor in late September, 2020.

Table 1 shows results of the four tests described in the methods section for each drug category. Overdose deaths after the pandemic began have increased substantially for all types of drugs, except for heroin, which decreased by half and for anxiolytics, which remained unchanged (see Table 1 Column Block 1, Number per month). There is a substantial change after April 2020 (Column Block 2 Immediate change); all drugs recorded increased levels after the pandemic began; the highest statistically significant increase involves alcohol and fentanyl while heroin and anxiolytics exhibit non-significant increases. We find no significant difference in slopes (Column Block 3) during the pandemic through 2021, compared to before, except for anxiolytics, where there is a significant reduction. Comparing the fitted values of December 2021 to that just prior to the pandemic, we find significantly higher overdoses among all categories, except for heroin, which saw a significant reduction, and for anxiolytics, which showed no significant difference (Column Block 4). The largest increases were due to alcohol, amphetamines, cocaine, and fentanyl.

**Table 1:** Comparison of deaths caused by overdose before, at the start, and during the COVID-19 pandemic.

**Category**	**Number per month**			**Immediate change**			**Monthly slope after vs. before**			**Ending to just prior to pandemic**		
	**Before** ^*^	**Since** ^**^	**Signif**	**Ratio**	**95% CI**	**Signif**	**Difference**	**95% CI**	**Signif**	**Ratio**	**95% CI**	**Signif**
Overdose	40.9	51.2	0.0001	1.494	(1.23, 1.815)	0.0001	−0.011	(−0.032, 0.011)	0.33	1.272	(1.045, 1.55)	0.015
Opioids	29.5	38.6	0.0001	1.496	(1.195, 1.873)	0.00034	−0.019	(−0.044, 0.0067)	0.14	1.255	(0.998, 1.577)	0.047
Fentanyl	21.4	33.4	0.0001	1.783	(1.381, 2.303)	0.0001	−0.022	(−0.052, 0.0073)	0.132	1.45	(1.118, 1.881)	0.0043
Heroin	3.8	1.8	0.0023	2.076	(0.957, 4.501)	0.059	−0.049	(−0.14, 0.039)	0.265	0.221	(0.076, 0.645)	0.0048
Stimulants	17.8	26.6	0.0001	1.412	(1.061, 1.878)	0.016	−0.008	(−0.04, 0.024)	0.619	1.625	(1.226, 2.154)	0.00058
Amphetamines	8.5	15.8	0.0001	1.547	(1.041, 2.299)	0.028	−0.013	(−0.059, 0.033)	0.576	1.981	(1.343, 2.922)	0.00044
Cocaine	11.4	15.4	0.0001	1.448	(1, 2.096)	0.045	0.0058	(−0.035, 0.047)	0.775	1.606	(1.113, 2.317)	0.0098
Anxiolytics	10.3	11	0.09	1.316	(0.898, 1.93)	0.151	−0.05	(−0.094, −0.0055)	0.025	0.773	(0.515, 1.159)	0.204
Alcohol	5.9	10.3	0.0001	2.712	(1.628, 4.517)	0.0001	0.021	(−0.035, 0.076)	0.455	2.205	(1.315, 3.7)	0.0022

All tests are based on quasi-Poisson modeling using fitted values and their (correlated) standard errors. ^*^Before dates correspond to January 2019 to April 2020. ^**^After dates correspond to May 2020 to December 2021.

**Figure 3:**
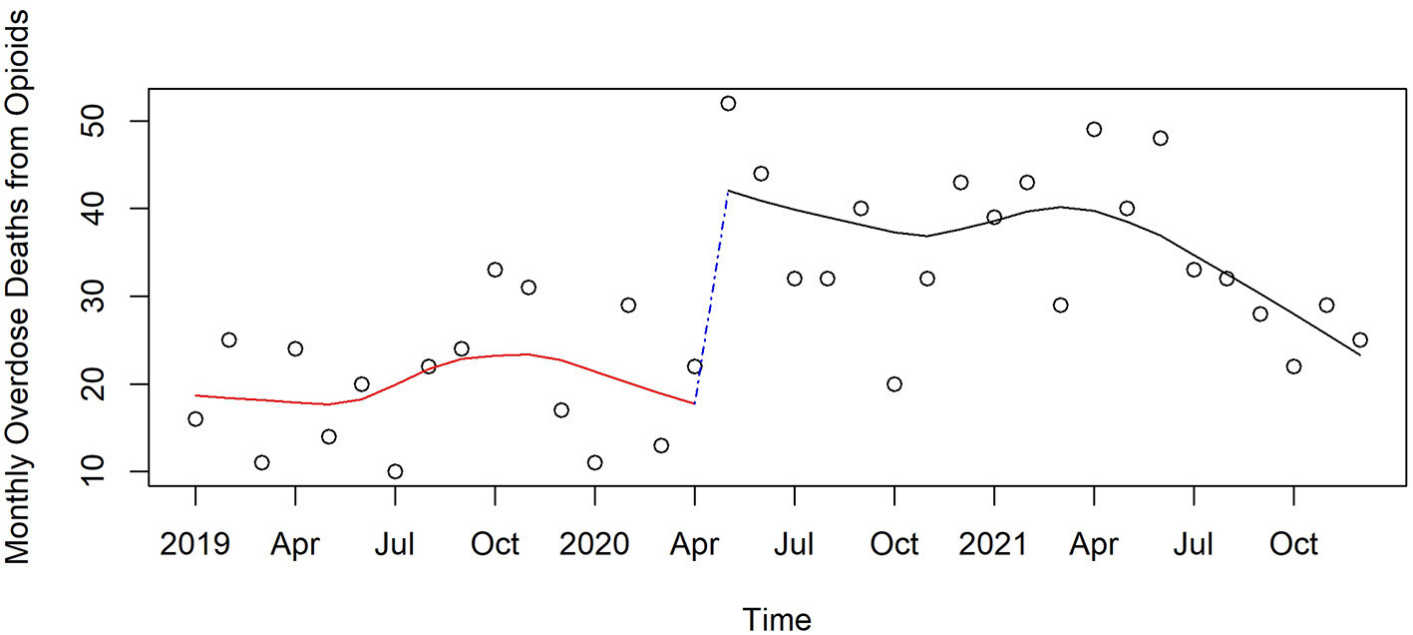
Monthly opioid deaths in Pinellas County before, at the beginning, and during the pandemic, 2019–2021.

**Figure 4:**
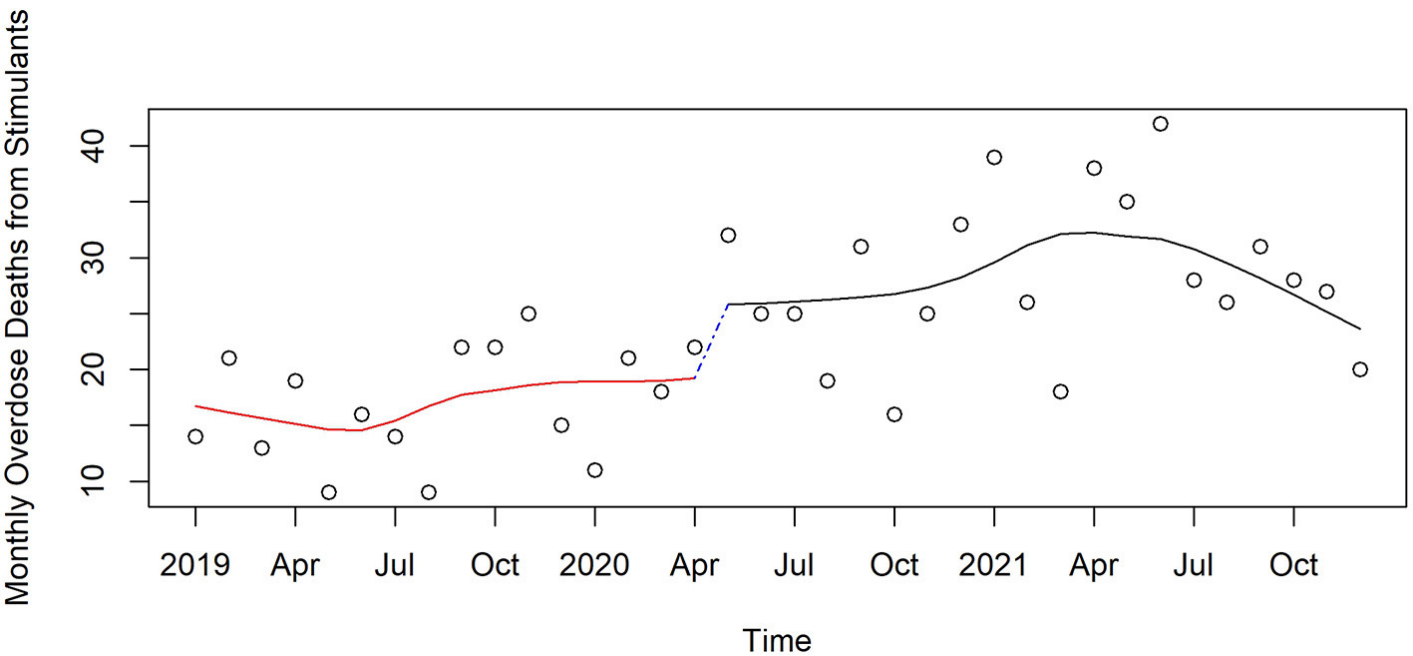
Monthly stimulant deaths in Pinellas County before, at the beginning, and during the pandemic, 2019–2021.

**Figure 5:**
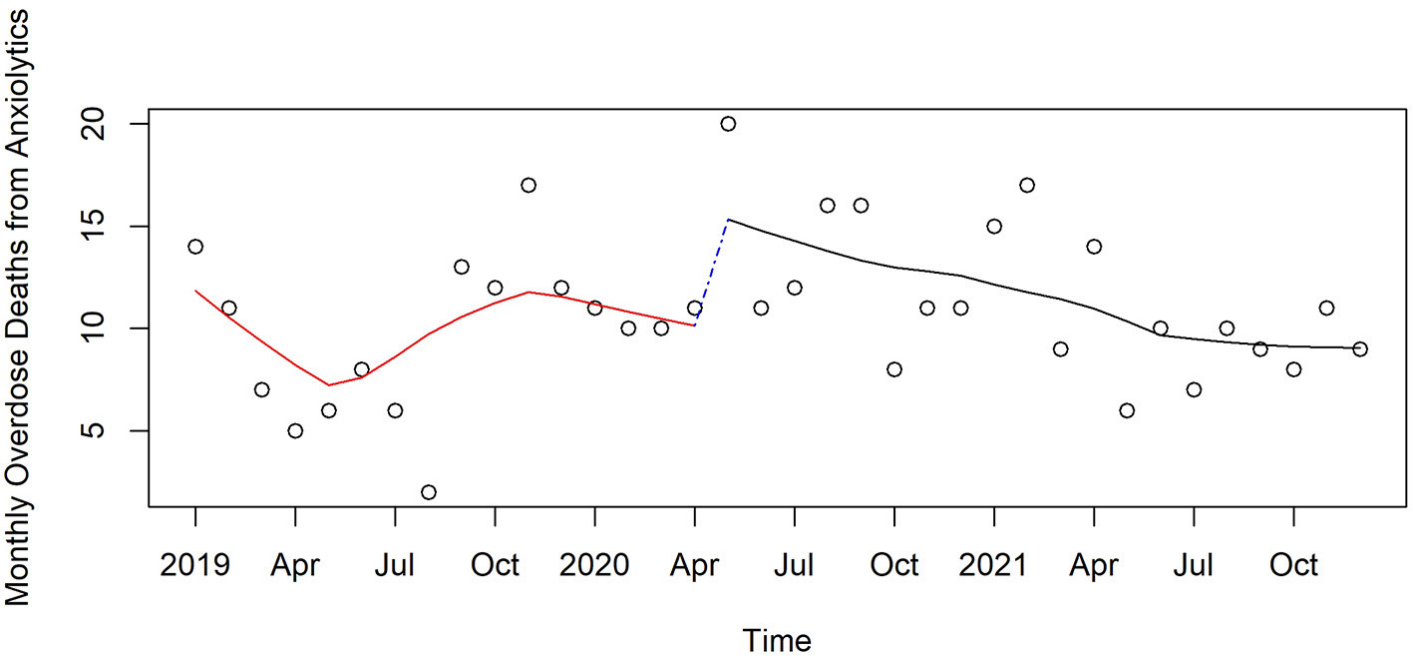
Monthly anxiolytic deaths in Pinellas County before, at the beginning, and during the pandemic, 2019–2021.

**Figure 6:**
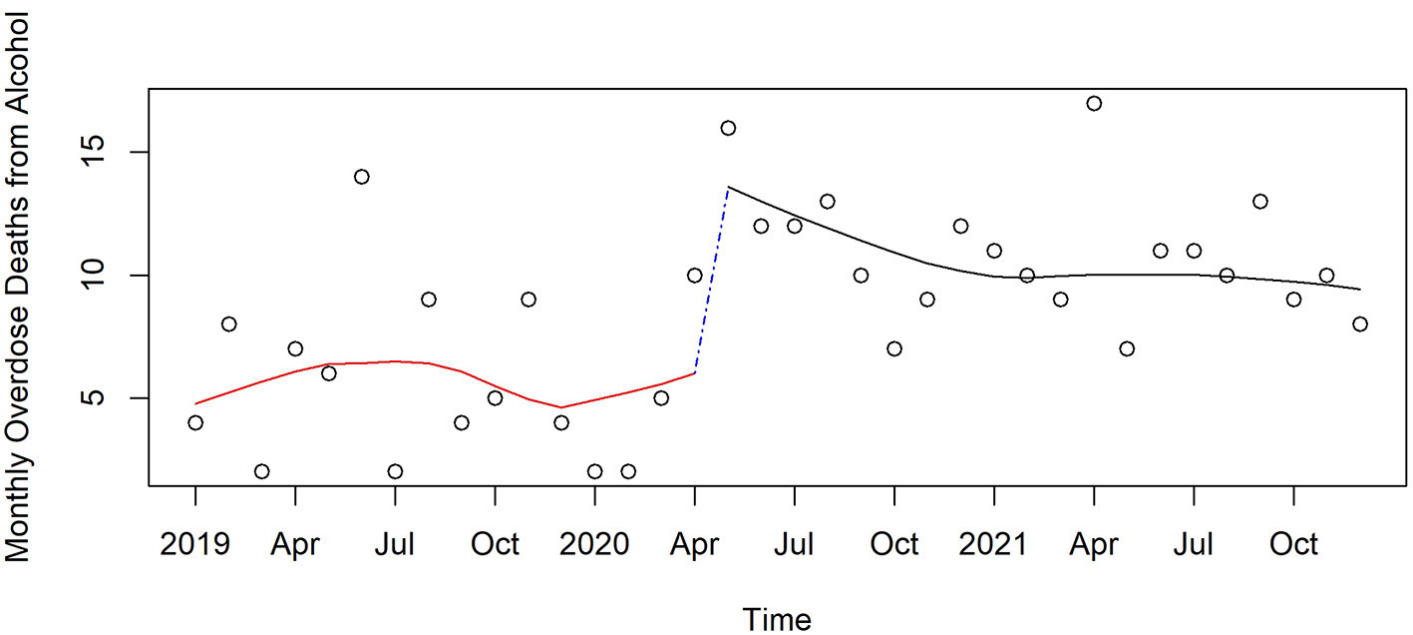
Monthly acute alcohol deaths in Pinellas County before, at the beginning, and during the pandemic, 2019–2021.

Figures 3–6 and Supplementary Figures 1–4 show counts of overdose deaths each month caused by specific drug sub-categories listed in Table 1. For overdoses (Figure 2), opioids [all (Figure 3), fentanyl (Supplementary Figure 1), and heroin (Supplementary Figure 2)], and anxiolytics (Figure 5), the highest number of deaths occurred in exactly the same month of May 2020. This month is also the maximum overdose death rate for the U.S, as shown in Figure 1. Another consistency across these drug categories was the consistent elevation of overdoses after COVID began over the pre-COVID period, which held for all drug categories except heroin.

As a fraction of the number of overdose deaths, the largest proportion is due to fentanyl – causal in 52% before the pandemic and 67% after the start of the pandemic (p - 0.01 by Chi-square test). The proportion of deaths caused by heroin decreases from 9% before the pandemic to 3% after the start (*p* < 0.0001). Both amphetamines and alcohol causal determinations increased at the start of the pandemic (21% to 31% for amphetamines. *p* < 0.001 and 14% to 20% for alcohol, *p* = 0.02), while proportions for the other drug categories remained similar before and after the pandemic.

#### Multiple drugs, fentanyl, and its analogs

Prior to the pandemic, fentanyl caused twice as many deaths as did the drug with the next highest cause of death – cocaine. After the start of the pandemic through December 2021, fentanyl-caused deaths rose an additional 50% and it was still twice as deadly as the two next most common drugs of amphetamine and cocaine. Fentanyl was causal in 66% of overdoses. Because the majority of deaths are associated with multiple drugs, we examined fentanyl presence among decedents, as a function of the number of other drugs that were detected by toxicology. Fentanyl by itself ***was never*** identified by toxicological findings among those who died from overdose. Only 8% of decedents used fentanyl along with one other drug; 17% had fentanyl with two other drugs; 25% three other drugs and 18% four other drugs. We also examined how prevalent fentanyl was in overdoses as a function of the total number of drugs across all toxicological findings. If two drugs were found, 36% of the time fentanyl was one of these drugs. For three drugs fentanyl was present 61%, and for four drugs found, fentanyl was present 75% of the time.

#### Overdose deaths from medications commonly used for opioid use disorder

Similar to other jurisdictions (8, 22), the number of overdoses in Pinellas County caused from drugs generally used to treat opioid addiction (e.g., methadone), was stable at a comparatively low rate 2 deaths per month throughout the 3 years, despite there being more “take-home” methadone during the pandemic.

#### Overdose by demographics

Over the 3 years, there were 1,528 White persons, 130 Black persons, 10 Asian persons and 33 persons whose race was classified Other/Unknown who died with a primary cause of overdose. White persons were overrepresented by 9% compared to their population proportion throughout the county, while Black persons and Asian persons were underrepresented by 31% and 84% respectively (χ^2^ = 80.7 on 3 df, *p* < 0.0001). Males were 2.27 times as likely as females to have died from overdose.

We examined whether there are any changes in the proportion of overdoses by White persons vs. Black persons and by males vs. females before and after the pandemic began and found no significant differences by these demographic moderators. Thus, although there was a 56% increase in overdose rate after the pandemic compared to before for White males (95% CI 3% to 137%, *p* = 0.04), this rate was not significantly different from non-White male persons or for females.

The median age of death among those with a primary cause of death due to overdose was 45, with half dying between 36 and 55. We found no significant difference in the increase in overdose deaths among those above or below 45.

### County level factors potentially influencing overdose deaths during the pandemic

Collectively, the county has diverse opportunities and resources that have the potential to impact overdose deaths. The major factors we examine here are organized in the following broad categories: changes in interdiction, rescues, addiction treatment, and social services, all of which potentially can impact overdose deaths.

### Interdiction

Our examination includes toxicologic findings of drugs seized by police and arrests for drug offenses.

#### Drugs seized by police

**Figure 7:**
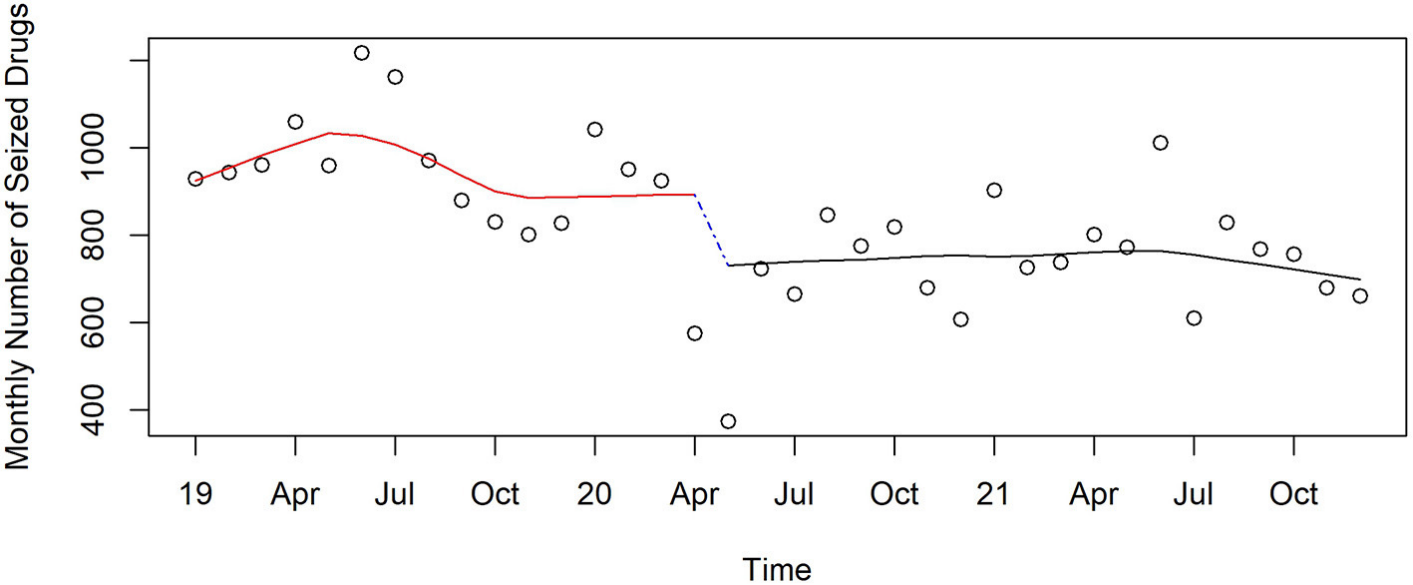
Monthly seizures of drugs in Pinellas County before, at the beginning, and during the pandemic, 2019–2021.

Drug seizures by police dropped precipitously in March and April 2020 then remained 20% lower than before the pandemic (Figure 7). This level of immediate reduction in seizures was observed for the following classes of drugs; opioids (29% reduction, *p* = 0.02), fentanyl (39%, *p* = 0.06), stimulants (39% reduction, *p* = 0.02), methamphetamine (42% reduction, *p* = 0.02), and cocaine (40% reduction, *p* = 0.004). For most of these drug categories, seizures then increased later in the pandemic but most often remained lower than before the pandemic. Seizures of cannabis, heroin, and benzodiazepines did not change significantly at the start of the pandemic.

#### Drug arrests

**Figure 8:**
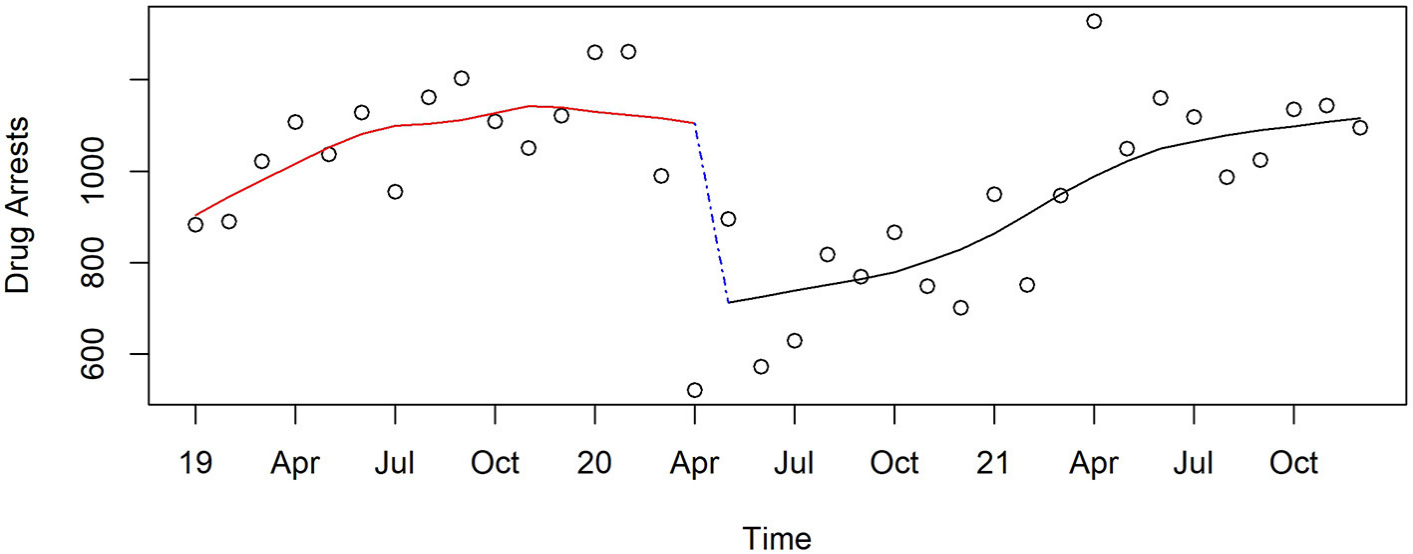
Monthly drug arrests in Pinellas County before, at the beginning, and during the pandemic, 2019–2021.

Drug arrests (Figure 8) dropped precipitously at the start of the pandemic (56% reduction, *p* < 0.0001 by quasi Poisson modeling) as police resources were diverted to pandemic related needs. They gradually increased over the course of the pandemic but had not quite reached the level of March 2020 (96% ratio, *p* = 0.02).

### Overdose rescue

**Figure 9:**
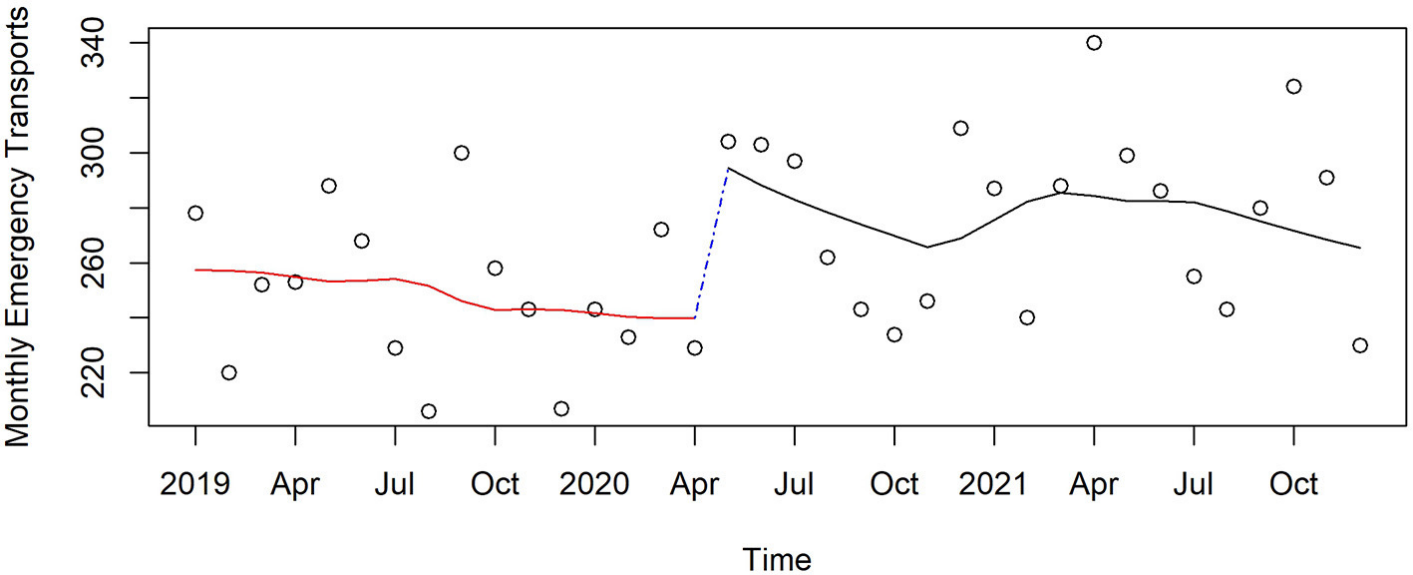
Monthly transport calls for suspected overdose in Pinellas County before, at the beginning, and during the pandemic, 2019–2021.

Emergency and police transport for suspected overdose remained stable in 2019 up to the start of the pandemic; it then increased by 12% (*p* = 0.01) from the transition month of April 2020, and remained 15% (*p* < 0.001) higher through the end of 2021 compared to before the pandemic began (Figure 9).

**Figure 10:**
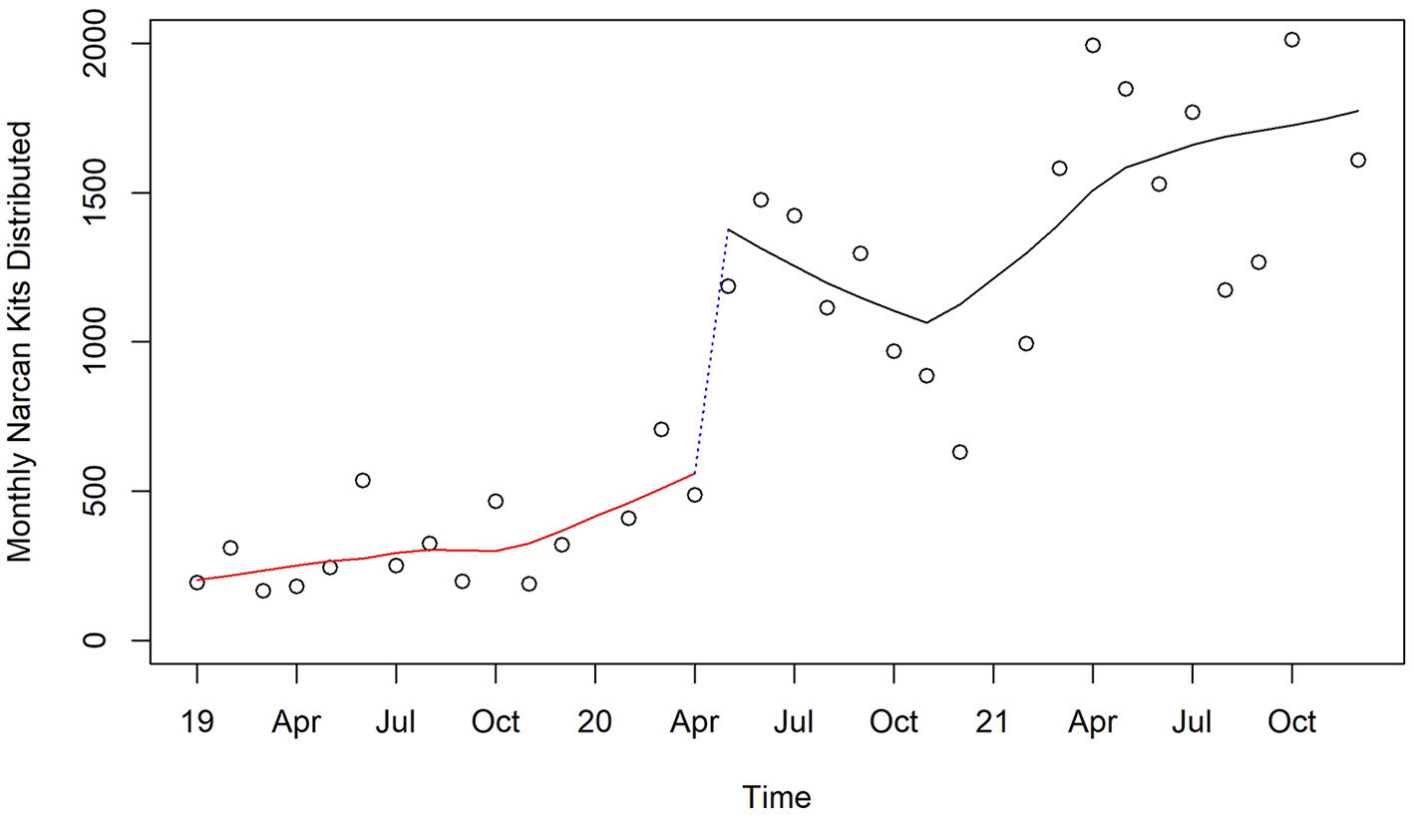
Monthly distribution of Narcan Kits by Florida Department of Children and Families in Pinellas County before, at the beginning, and during the pandemic, 2019–2021.

Increasing access to overdose recovery medications to community and business organizations was a priority for the opioid taskforce. Naloxone, in the form of Narcan kits, began to be more widely distributed in 2019 by the State of Florida Department of Children and Families in Pinellas County. Because of the way the state obtained Narcan kits at the end of two of the 3 years, there were extensive distributions in January 2020 (11,456) and January 2021 (20,296). As these are an order of magnitude larger than the distributions for all other months, we remove these two outliers to examine the remainder changes over time. Distribution was increased at the beginning of the pandemic by 93% (*p* < 0.0001) in the next month and continued at an increasing level through the study period, achieving at the end of 2021 377% (*p* < 0.0001) of the monthly distribution compared to that just before COVID began (Figure 10).

**Figure 11:**
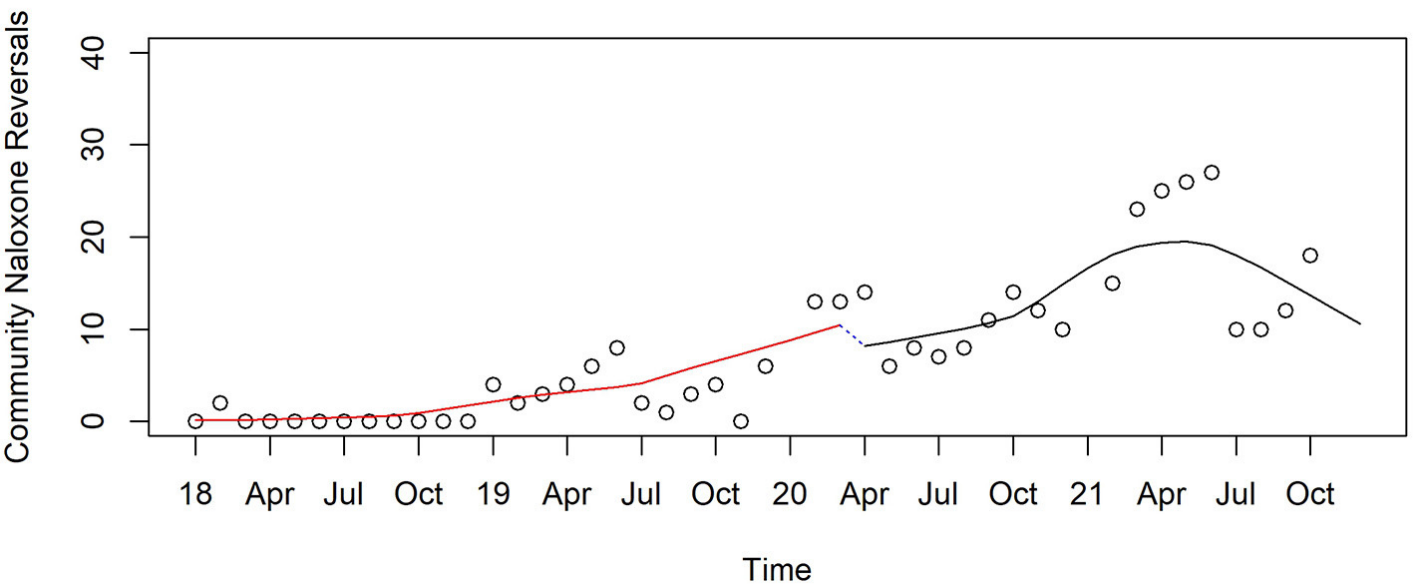
Monthly naloxone reversals reported by communities in Pinellas before, at the beginning, and during the pandemic, 2019–2021.

Counts of known naloxone rescues reported by community organizations receiving Narcan kits increased steadily before and during the pandemic, with a non-significant drop between April and May 2020 followed by an increase through April 2021 and a decrease for the rest of the year, with no significant change from the end of 2021 compared to April 2020 (Figure 11).

### Opioid medications and treatment for opioid and other drugs

#### Methadone treatment

**Figure 12:**
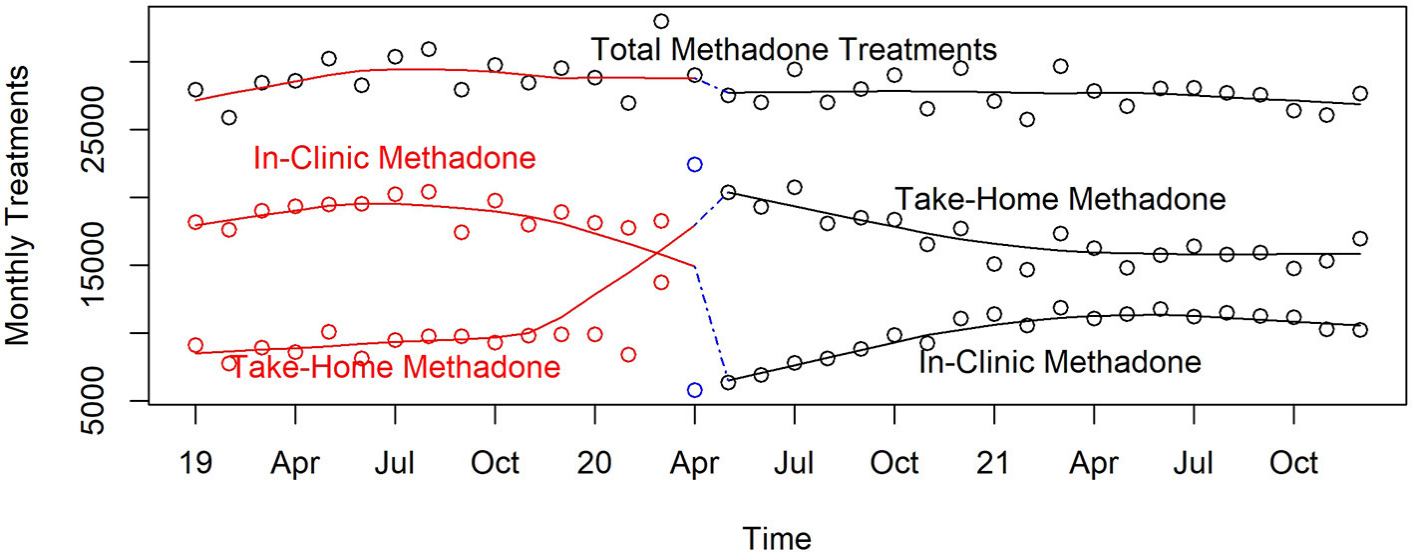
Monthly in-clinic and take-home methadone treatments in Pinellas County's public OTC, 2019–2021.

In the county's non-profit OTC, all treatment for OUD, as well as methadone treatment specifically remained relatively stable throughout the 3 years, except for a 6% reduction (*p* < 0.0001) at the beginning of the pandemic (shown in the top smoothed pattern in Figure 12). By the end of 2021, the number of methadone treatments was 90% of that before the pandemic began (*p* < 0.0001). This figure also shows the major difference between in-clinic and take-home methadone treatments over time. Prior to the pandemic the proportion of in-clinic methadone treatment was twice that for take-home methadone; this reversed immediately in the month afterwards and eventually equilibrated with 60% higher rates of take-home than in-clinic.

**Figure 13:**
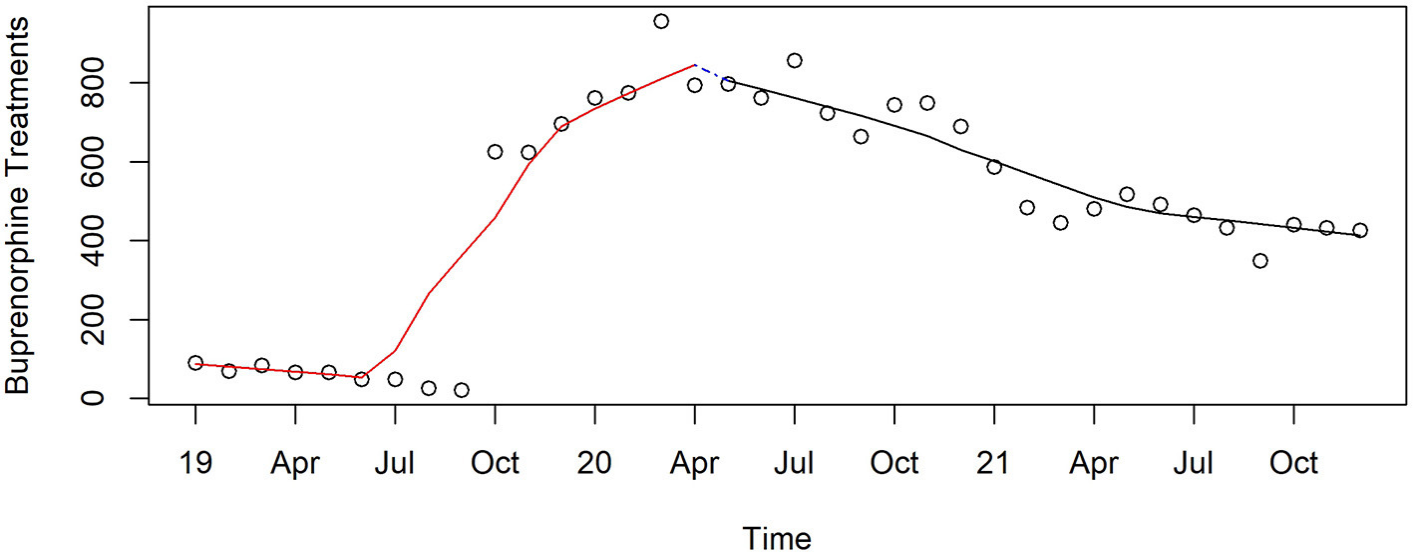
Monthly buprenorphine treatments in Pinellas County's public OTC, 2019–2021.

Buprenorphine was infrequently used by the county's public OTC before October 2019 when its use jumped to over 600 per month and rose to 800 when COVID first appeared (Figure 13). There was no abrupt change with the onset of the pandemic (non-significant), but its use dropped significantly through the end of 2021. Buprenorphine was provided half as often in this OTC compared to that at the start of the pandemic (*p* < 0.0001).

#### Counseling services for addiction

**Figure 14:**
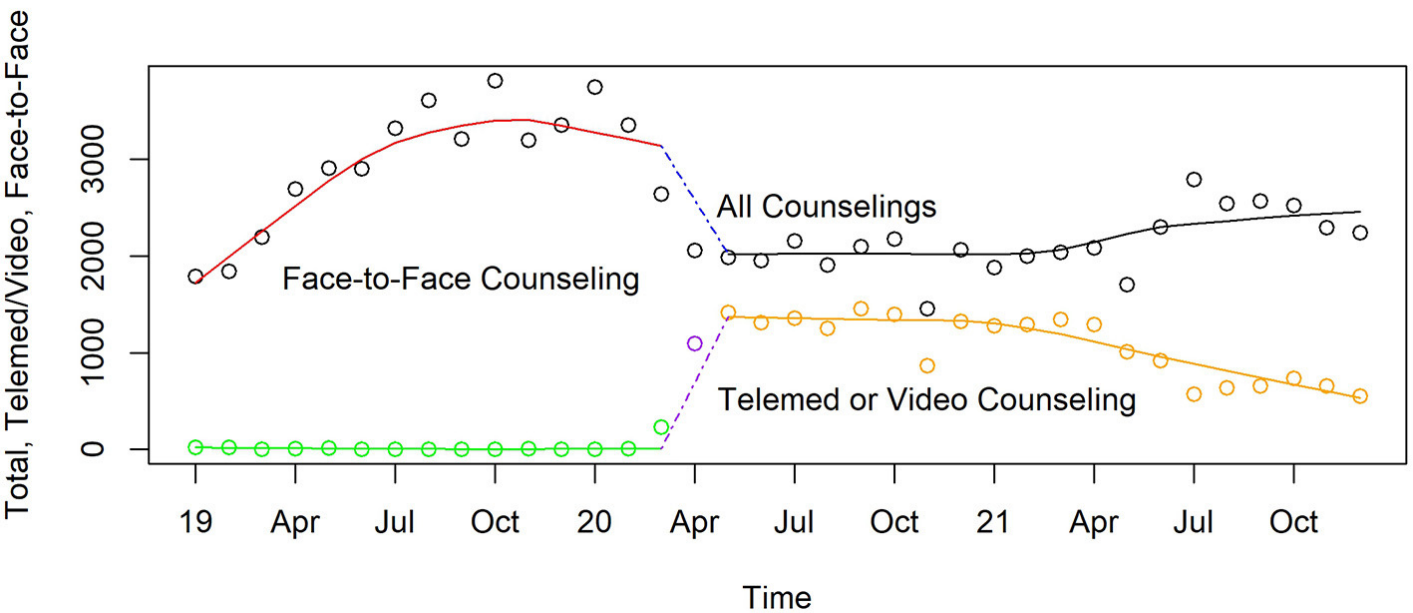
Monthly face-to-face and telemed/video counseling in Pinellas County's non-profit OTC, 2019–2021.

Total behavioral counseling services in the public OTC, shown in the top smooth curve in Figure 14, were rising before the pandemic; then flattened out until just before the pandemic appeared. Total behavioral counseling services then decreased 38% (*p* < 0.0001) at the beginning of the pandemic, then stayed relatively constant through 2021 where it remained 20% (*p* < 0.0001) lower than at the beginning of the pandemic. There was a major shift in the way that counseling was delivered during the pandemic. Before the pandemic, all services were face-to-face as shown in Figure 14, but immediately after the pandemic began 75% of all counseling used telehealth. By the beginning of 2021 face-to-face counseling began to replace the telehealth modality. By the end of 2021 two-thirds of counseling services were face-to-face.

#### Opioid medication and treatment prescriptions

According to prescription drug monitoring reports, the number of opioid medications prescribed for either pain or addiction treatment decreased by 7% by the end of the second quarter of 2020 (238,000) compared to the number of prescriptions 1 year earlier (256,000). Also, the number of patients served with these prescriptions went down 13% (72,000 from 83,000 a year earlier). At the level of data available, we cannot assess whether the change was greater for addiction or pain treatment.

### Social services

#### Homelessness

Because of the pandemic, the annual Unsheltered Homeless Count did not take place in 2021. This plus the lack of accurate homeless data from jails and schools and lack of quarterly breakdowns makes it difficult to assess how homelessness changed during the pandemic. Nevertheless, existing data (23) consistently find the number reported who were homeless decreased 10% in 2020 (2,209) compared to the previous year (2,415) (24). This reduction fits with the efforts of the county to pay for housing during COVID using hotel rooms that were empty because of the extreme reduction in tourists starting in April 2020 (down 90% from the previous month and year) through February 2021 (down 23% from the previous year) (25). Evictions from rentals that were federally funded or used federally guaranteed loans were halted in March, 2020 by the Coronavirus Aid, Relief, and Economic Security Act on March 27, 2020 (26), and further evictions were prohibited by the CDC from September 1, 2020 through the study period (27). We also examined whether there was an expansion or contraction of federal health care over this time period. While Medicaid enrollment in Pinellas County is low compared to other Florida counties and the nation, there was a 10% expansion in enrollment in September 2020 (16.1%) compared to the previous year's rate (14.6%).

#### Federal financial relief

Economic impact payments began in March, 2020 ($1,2000 per adult, $500 per child), followed by December, 2020 ($600 per adult and child), and March 2021 ($1,400 per adult and child) (26).

### Summative modeling to examine factors related to overdose deaths

Table 2 presents single predictor (column 1) and multiple regression (columns 2 and 3) type time series relationships between monthly changes in interdiction, treatment, and rescue and overdose deaths. We report findings from Poisson-time series modeling as the fits with negative binomial modeling were slightly poorer based on Bayesian Information Criterion (BIC) (28) but differed little in their coefficient estimates. While all predictor variables except drug arrests were individually related to concurrent month overdose (column 1), only drug seizures and 911 transport were significantly related when all variables were included (column 2). Regarding changes over time related to COVID, the simultaneous predictors showed higher rates after compared to before COVID appeared in the county, and a modest drop over time after COVID appeared. No interaction terms involving before and after COVID were significant (not shown in Table 2). When using backward elimination only two variables remained significant (column 3 of Table 2). Drug seizures remained highly significant, with fewer overdoses when seized drugs were high. Secondly, in contrast to what we might expect, there remained a highly significant positive relationship between 911 transports and overdoses (trimmed model in column 3). It is very likely that a positive feedback loop exists where transports increase when more overdoses occur, so this positive coefficient may arise from that relationship.

**Table 2:** Univariate and multiple predictors of overdose counts Pinellas County 2019–2021.

	**Single predictor**	**Simultaneous predictors**	**Trimmed model**
**Interdiction**	Beta (se) p-value	Beta (se) p-value	Beta (se) p-value
Seized drugs	−0.957 (0.184) < 0.001^***^	−0.805 (0.313) 0.01^**^	−0.691 (0.115) < 0.0001^****^
Drug arrests	−1.315 (1.235) 0.29	0.504 (2.547) 0.84	
**Treatment**	Beta (se) p-value	Beta (se) p-value	
Methadone	−0.065 (0.016) < 0.001^***^		
In-clinic methadone	−0.035 (0.008) < 0.00001^***^	0.00 (0.015) 0.99	
Take-home methadone	−0.029 (0.007) < 0.01^**^	−0.020 (0.014) 0.16	
**Rescues**	Beta (se) p-value	Beta (se) p-value	Beta (se) p-value
911 transports	3.239 (0.754) < 0.01^**^	2.86 (1.02) < 0.01^**^	2.800 (0.504) < 0.0001^****^
**Time**	Beta (se) p-value	Beta (se) p-value	
After COVID	0.55 (0.089) < 0.00001^****^	0.60 (0.23) < 0.01^**^	
Linear	0.008 (0.006) 0.19		
Linear before COVID	−0.039 (0.007) < 0.001^***^	0.013 (0.009) 0.16	
Linear after COVID	0.018 (0.003) < 0.001^***^	−0.011 (0.007) 0.10	

Columns: Single Predictor column uses univariate analysis of each measure; Simultaneous Predictor includes all predictors in the model while Trimmed Model includes only terms whose p-values were < 0.1 from a backwards elimination procedure. Analyses: All analyses were run using time series [ARMA (1, 1)] Poisson modeling off monthly overdoses for years 2019–2021. Predictor Variables: All Interdiction, Treatment, and Rescue variables are measured in counts per month/1000. Seized Drugs is a count of all Opioid, Stimulant, or Benzodiazepine seizures thus leaving out cannabis and other less toxic drugs. Methadone counts are the sum of In-Clinic and Take-Home, so only the latter two are presented in the Multiple Predictor column. After COVID is a contrast between May 2020 through 2021 vs. pre-COVID months (2019 through April 2020). Linear and Linear Before COVID covariates are based on month number since 2018, Linear After COVID starts May 2020.

Significance: ^*^p < 0.05, ^**^p < 0.01 ^***^p < 0.001 ^****^o < 0.0001.

We conducted additional regression analysis to examine the stability of the two backward elimination findings further; instead of using the same month of transports to predict overdose at the same month, we used the previous one- and two-months' transports, to see if the relationship changed. For seized drugs predicting overdoses 1 month later, the coefficient became stronger, from −0.691 to −0.93, SE = 0.218, *p* < 0.0001.

For 911 transports predicting overdoses 1 month later, we found the relationship diminished only slightly from a coefficient of 2.8 for no lag in Table 2 column 3 vs. 2.5, SE = 0.85, *p* < 0.01.

## Discussion

*There is a substantial increase in overdose deaths at the start and during the pandemic, as compared to the pre-pandemic period. An abrupt increase occurs at the same time as COVID-19 deaths and shutdowns began, with a moderate decline through 2021 but remains elevated over 2019 counts*. The immediate impact of the pandemic manifested in increased deaths from all drug categories except anxiolytics, and were particularly high for alcohol, fentanyl, amphetamine, and cocaine. These increases began when treatments, services, and enforcement activities were curtailed due to the pandemic. However, they also persisted over the next 20 months as disruptions in the availability of social and medical services continued, but EMS rescues with naloxone continued to increase at a higher rate than they did pre-pandemic. Increasing overdose trends during the pandemic have been reported in other studies (29–33), and the pandemic's immediate increase was shown in Ohio (30).

*In Pinellas County overdose decedents, fentanyl and its analogs were never found alone, but always in the presence other drugs*. Fentanyl and its analogs causally contributed to two-thirds of overdose deaths.

Stimulants (amphetamines and cocaine) were causal in 49% of overdoses, often in combination with opioids. The number of drug overdose deaths where alcohol was a causal factor doubled from the pre-pandemic period, a greater percent increase than any other drug.

*No increase in methadone-related deaths is seen during this pandemic period despite major relaxing of policy changes regarding take-home methadone doses during COVID* *(**8**)* *and its consequences* *(**22**)*. This finding supports previous studies (8, 22, 34).

*Compared to their population rates, White persons are more likely to die of overdose than are Black persons and males more than females. Both Black persons and White persons are more likely to die from overdose during the first year of the pandemic than before*. Elsewhere we have noted that Black persons in the Florida Medicaid population are much less likely to be referred for opioid treatment than White persons, which translates into higher all-cause mortality (35).

*With a median age at death of 45 and half aged 36 to 55, those dying of overdose are primarily middle-aged*. There are no detectable differences in overall or specific drug deaths between those older or younger than 45 before, at the beginning and during the pandemic. However, their similar rates of increase do not necessarily imply the dynamics are the same as risk factors may differ by age.

*In the midst of the major disruptions in societal and health care functioning at the onset of the pandemic, opioid medical and behavioral treatment systems as well as EMS and community rescue systems adapted rapidly and effectively*.

Treatment remained more difficult to obtain due to policies and procedures that limited contact during the pandemic, including quarantine protocols, continued requirements for in-person physicals for new patients for methadone, and unemployment payments higher than the average salary for some clinic staff leading to staffing shortages. The data from the county's only public OTC showed that opioid treatment availability suffered briefly because of the lack of masks and other protective equipment as well as quarantines and hiring challenges. However, there was a very rapid shift to take-home methadone. If another pandemic crisis arose, having a supply of PPE on hand would mitigate some of this supply challenge. Other immediate and longer-term disruptions in access to behavioral counseling, social and medical services (e.g., hospitalization) occurred during this time period.

Not all service access decreased, however. Importantly, housing for the homeless increased in 2020 in response to the increased risk of COVID infections, and EMS rescues abruptly increased at the onset of the pandemic. Reported community rescues with naloxone remained stable at the pandemic's onset and throughout the entire 3 years.

We note that increased overdose deaths began at the start of the pandemic when there was a drop in in-clinic methadone, cessation of new patient admissions for methadone, a shift to take-home methadone and a reduction in behavioral counseling, especially face-to-face. Immediate disruptions in available and accessible drug treatment programs, increased social isolation and related changes in drug use behavior (using alone, for example), and reduced drug seizures by police at the start of the pandemic, all may have contributed to the immediate rapid rise in overdose death rate. Enforcement of drug laws took a back seat to addressing pandemic related crisis with seizures and arrests falling dramatically at the beginning of the pandemic. Other studies have suggested that increased enforcement is related to increased overdose deaths, but our analysis suggests that decreased enforcement was an antecedent to increased overdose deaths in the specific case of the pandemic (36).

### What policy decisions are suggested by these data

The Health and Human Services Overdose Prevention Strategy identifies four pillars: primary prevention, harm reduction, treatment, and recovery. Recognizing that the boundaries of these pillars are at times overlapping, we present opportunities in these four categories, starting with treatment, the most direct pillar to overdose. We include opportunities suggested by these data suitable at the onset of a pandemic as well as in the later refractive months as functioning of systems begins to return. While the onset and refractive period of this particular pandemic has passed, a wide range of natural or human disasters could provoke similar systemic effects. Thus, these opportunities may have relevance to future crises as well.

### Treatment

By sheer numbers, those in treatment declined precipitously at the onset of the COVID pandemic. Access to MOUD (37), which can reduce overdose deaths by a factor of ten, remains limited due financial, workforce, linkage, and logistical challenges (38), all of which were accentuated during the pandemic. Expanded medical and public health efforts are warranted given the increase in fentanyl and elevated overdose deaths 1 year beyond the pandemic's start.

Treatment centers were unable to secure masks and other protective equipment and had major difficulties maintaining sufficient staff. Were another pandemic or human or natural disaster to occur, the situation would be repeated unless there was better preparedness in addiction treatment centers and their sources of new patients, such as jails, hospitals, and trusted community organizations. Now that we are aware of the logistic, policy, and social determinant barriers that occurred with the pandemic as well as facilitators including take-home methadone that worked well, and the expanded service network of buprenorphine prescribers, we suggest immediately focusing on increasing MOUD treatment supply as well as demand. In both supply and demand, primary care physicians may provide one of the few contacts that patients have with the medical system during a pandemic, either in-office or through tele- or video-medicine, and are therefore positioned to educate, treat, or refer those misusing substances (39). Recent changes to federal law removing the buprenorphine waiver training requirement to prescribing this drug has the potential for greatly expanding and continuing long-term treatment (40), especially in rural and underserved areas, but more needs to be done to overcome resistance of non-waivered providers to screen for opioid misuse and to prescribe (41). Another important issue is that the slow-to-change public and private reimbursements for specialty addiction treatment make it difficult to hire staff when salaries in other businesses are increasing rapidly.

### Prevention and harm reduction

The majority of those who are dependent on opioids are not in treatment and some of those who have overdosed on opioids may not be dependent on opioids but are unfortunate recipients of another drug class contaminated with opioids like fentanyl. At the beginning of a pandemic there may be interruptions in their regular supply leading to greater risk taking, and greater alcohol consumption and mixing of drugs. Intensified harm reduction strategies at the beginning of a crisis like the pandemic may save lives. Public health approaches to reduce overdose deaths include safe injection sites and other means of ensuring drug users are not alone when they use (42), fentanyl and other drug test strips so that users are aware of the content of the drugs they are ingesting (43, 44), community distributed and leave-behind naloxone for those rescued (45), and suboxone for high-risk individuals, such as those who are exiting from jails (46, 47). Wider use of several of these harm reduction approaches, such as testing for contamination of street drugs with fentanyl strips, and the county's newly begun integrated public health services with syringe exchange, could reduce the dangers of street drugs.

It is noteworthy that in our summative analyses, the number of 911 rescues is found to be positively related to overdose deaths. We interpret this directional effect as suggesting that the number of rescues is more likely to increase in response to both non-fatal overdose increases as well as fatal overdoses. Such a directional relationship is commonly seen in complex systems behavior where such feedback occurs.

Regarding prescription opioids, a cluster of policies and regulations has been developed in an attempt to balance the dual objectives of providing pain relief and reducing misuse and causing addiction. These policies and regulations may work well under normal circumstances, but they may be inadequate in a crisis situation such as a pandemic. As Florida has a history of hurricanes that strain prescription access, there exists policy changes in opioid access that could be applied in other types of crises like a pandemic.

Placing some limits on alcohol sales rather than expanding access to alcohol as was done in most states during the COVID-19 pandemic, is recommended.

While enforcement of drug laws has received negative attention in recent years, our analysis hints that reduced enforcement of drug laws was one of several proximal factors that may be related to the rapid increase in overdose deaths at the beginning of the pandemic. Our summative analyses suggest that the reduction in seized drugs that occurred during COVID may have led to an increase in overdoses.

### Recovery

It is well known that among those in recovery, experiencing higher numbers and more intensive stressors, which routinely occurs at the onset and during a disaster, can lead to relapse (48). It is recommended that community organizations respond by providing additional outreach using peer and professional support, as well as having the local government address needed social determinants. Housing for the homeless, increased access to emergency medical care and financial support from federal Medicaid and unemployment payments may have softened the effect of lack of enforcement, outreach and treatment services created by the pandemic.

## Limitations

There are several limitations to this study. This paper addresses changes occurring during the pandemic in one county; other counties' exposure and response to the pandemic may differ. The full impact of drugs on mortality is undercounted as we restricted the definition of overdose, excluding those whose manner of death is natural. New toxicologic testing for emerging drugs such as xylazine (49), led to small changes in record keeping for overdose that occurred during the study period. We do not know how many users had the clear intention to use fentanyl, or whether it had been added without user awareness. This paper does not fully examine releases from jail or linkage to service systems or their lack thereof (e.g., homelessness, job loss). Treatment data is from one organization and may not reflect the experience of other organizations in the county.

As mentioned in the introduction, we believe the methods are appropriate for diverse counties to use but remain cautious in considering some these findings from a single county to be reflective of other counties. The pattern of overdose deaths in Pinellas County does share similarities with US national trends during the pandemic but shows differences as well. Regarding similarities, in particular, Figure 1 shows that the monthly number of overdose deaths in the US; deaths increase from the beginning of 2018 and peak in May 2020. Pinellas County overdose deaths across multiple drug categories also peak in May. The national overdose mortality rate drops soon thereafter and then rises precipitously; for Pinellas County overdose deaths began to decline slightly through the end of 2021. There was a quantitative difference in the national ratio of excess overdose deaths to excess non-COVID-19 deaths, which is 0.16, while that for Pinellas County, 0.28, exceeds that of 75% of other counties. Other local studies have reported similar increases. For example, a similar immediate increase at the start of the pandemic was shown in Ohio (30) while increasing trends after the pandemic began have been reported in other studies (29–33). These differences are what make local data analysis so important for decision making in addressing overdose deaths.

We recognize our analyses behind this data-driven decision-making approach do not provide a complete picture of all the factors affecting overdose mortality. Changes in access to and delivery of MOUD, drug seizures, drug arrests, releases from jail, availability of contingency management for stimulants, social services, and community organizational responses during the pandemic can all play independent or synergistic roles on overdose mortality. Policies to reduce overdoses can be informed somewhat by statistical analyses using time dependent observational data, such as those used here. Our analysis of multiple factors' concurrent relationship with overdose found a consistent and strong negative relationship between the number of seized drugs and overdose deaths, both concurrently and using a month lagged analysis. This finding contrasts with that reported in Marion County, Indiana, essentially after COVID arrived, where the authors conclude that seized drugs disrupted existing markets and led to more risky drug use and higher overdoses 3 weeks later (36). We also report a positive effect between 911 transports and later overdose. This counterintuitive relationship is likely a consequence of a bidirectional relationship, positive feedback where more overdoses increase transport while transports have negative feedback on overdose deaths. Given the positive relationship, it is indicative that more harm reduction strategies, including extensive leave-behind Narcan kits and training of household members by EMS could lead to reductions in overdoses. At the same time, we recognize these classic statistical modeling approaches are limited in predicting which combinations of interventions and implementation strategies would most reduce overdose deaths.

We note again that deaths from methadone overdose were not changed during this 3-year period. We are not able to determine whether methadone causing these deaths was supplied by treatment facilities or as street drugs.

### Three components of a model-driven decision support to reduce overdose deaths

A comprehensive decision support system should strive to answer three questions. First is what has happened, i.e., what is the pattern of overdose deaths by time. Second is why did these changes happen. Third, what would likely happen if alternative policies were enacted. The approaches in this paper represent answers to what happened and reasonable conjectures to why they happened. They have limitations in answering what-if future investments were made. In this section we describe an approach to answering this third important question.

Given the recent investments by the federal government aimed at reducing opioid overdose and the funding supplied to states and counties from legal agreements with the opioid manufacturers and distributors, there is an unprecedented opportunity to scale up and sustain opioid treatment and recovery services (50). We need to use these funding opportunities wisely. We recommend that decision makers take advantage of an integration of three components. The first involves listening to diverse community values, priorities, as well as strategies and tactics needed for making and carrying out decisions to address overdoses. Not only are community members an important source of knowledge about the local drug scene, but many can also identify and support strategies to reduce overdose deaths that reflect local values, histories, resources, and political realities. Secondly, local epidemiologic, toxicologic, interdiction, and service data, such as that described in this paper, needs to be used to inform decision making. These data help identify groups who are most affected, social, economic and policy changes that may be related to changes in death rates, and potential interventions. As a third component that can guide overdose intervention policies, we recommend partnering with community leaders by using simulation modeling of complex systems to distinguish which programs and policies are likely to make the most substantial impact. It does no good and saps a community's strength to deliver an evidence-based program that has minimal effect or cannot be well implemented due to local factors. Complexity modeling using, for example, agent-based modeling has been found to be valuable in shaping local HIV policies (51, 52) and could be helpful in guiding local medical and community decision making regarding overdose prevention as well (53). In contrast to the traditional data-driven decision support approach, which examines each intervention singly, agent-based models are able to evaluate the synergistic effects of multiple interventions delivered simultaneously with varying intensities and resource allocations, and provide comprehensive, systemic level predictions. Inexpensive simulation modeling can also take into account actual, anticipated, or contemplated changes in policies or planning, to allow comparisons of different intervention implementation strategies, such as comparing mobile vs. brick-and-mortar programs and can anticipate changes in the environment such as contamination of drug supply with new agents.

## Conclusion

Much of our data show that the prima facia availability of services after the abrupt appearance of a pandemic, while disrupted, can be (1) re-established, as with drug arrests, (2) replaced, as with take-home methadone for in-clinic, or (3) even accentuated, as we saw with housing, distribution of Narcan kits, and emergency rescues. However, availability of such services did not reduce fatal and non-fatal overdoses which increased immediately and remained higher through 2021 than before the pandemic. While systems including law enforcement were not as able to immediately adapt, lessons learned from their slower adaptation process could be applied in future crises. Most of the difference in adaptation rates relates to the need for policy, regulatory and funding system changes out of control of the direct actors in the county.

There remain significant pandemic-specific factors (e.g., isolation), societal structural barriers that limit access (e.g., transportation for and payment of MOUD), users' lack of knowledge and trust in service systems, as well as stigma that all limit utilization even as the systems involved move toward homeostasis. In Pinellas County as well as nationally, we have seen that these new equilibrium states fail to lower overdose deaths.

We recognize the relevance for decision support of data dashboard overdose relevant data. Such data may be the only way to document service changes and disparities that result from a major disruption such as a natural- or human-caused disaster. At the same time, standard statistical analyses of dashboard data such as we have presented here, have finite ability to examine systemic interactions. Other model-based approaches that use simulation could add to our understanding. In combination with Pinellas County's ongoing work of its Opioid Task Force, an integration of the three components of community partnership, local data, and agent-based modeling is now being developed. Future work will examine how acceptable and usable model-driven decision support turns out to be.

## Data availability statement

The original contributions presented in the study are included in the article/Supplementary material, further inquiries can be directed to the corresponding author.

## Ethics statement

The studies involving humans were approved by University of South Florida Institutional Review Board. The studies were conducted in accordance with the local legislation and institutional requirements. Written informed consent for participation was not required from the participants or the participants' legal guardians/next of kin in accordance with the national legislation and institutional requirements.

## Author contributions

CB: Writing—review & editing, Writing—original draft, Visualization, Validation, Resources, Project administration, Methodology, Funding acquisition, Formal analysis, Data curation, Conceptualization. KJ: Funding acquisition, Formal analysis, Writing—review & editing, Validation, Supervision, Resources, Project administration, Investigation, Data curation, Conceptualization. HH: Writing—review & editing, Validation, Investigation, Conceptualization. WV: Formal analysis, Writing—review & editing, Software, Resources, Methodology, Investigation, Conceptualization. DC: Writing—review & editing, Validation, Supervision, Resources, Project administration, Investigation, Funding acquisition, Conceptualization. JB: Writing—review & editing, Validation, Resources, Investigation, Conceptualization. RN: Writing—review & editing, Validation, Resources, Methodology, Investigation, Data curation, Conceptualization. TB: Writing—review & editing, Supervision, Resources, Project administration, Investigation, Funding acquisition, Data curation, Conceptualization. WP: Writing—review & editing, Supervision, Resources, Project administration, Investigation, Data curation.
