# Supplementary material for: Overdose deaths before and during the COVID-19 pandemic in a US county
Source: Front Public Health. 2024 May 27;12:1366161. doi: 10.3389/fpubh.2024.1366161 (PMC11163089; doi:10.3389/fpubh.2024.1366161)

Supplemental Figures for "Overdose Deaths Before and During the COVID-19 Pandemic in a US County."

These figures provide monthly overdose counts by Specific Drugs in Pinellas County Before, At the Beginning, and During the Pandemic, 2019-2021

Supplement Figure 1. Monthly Overdose Deaths Caused by Fentanyl in Pinellas County Before, At the Beginning, and During the Pandemic, 2019-2021

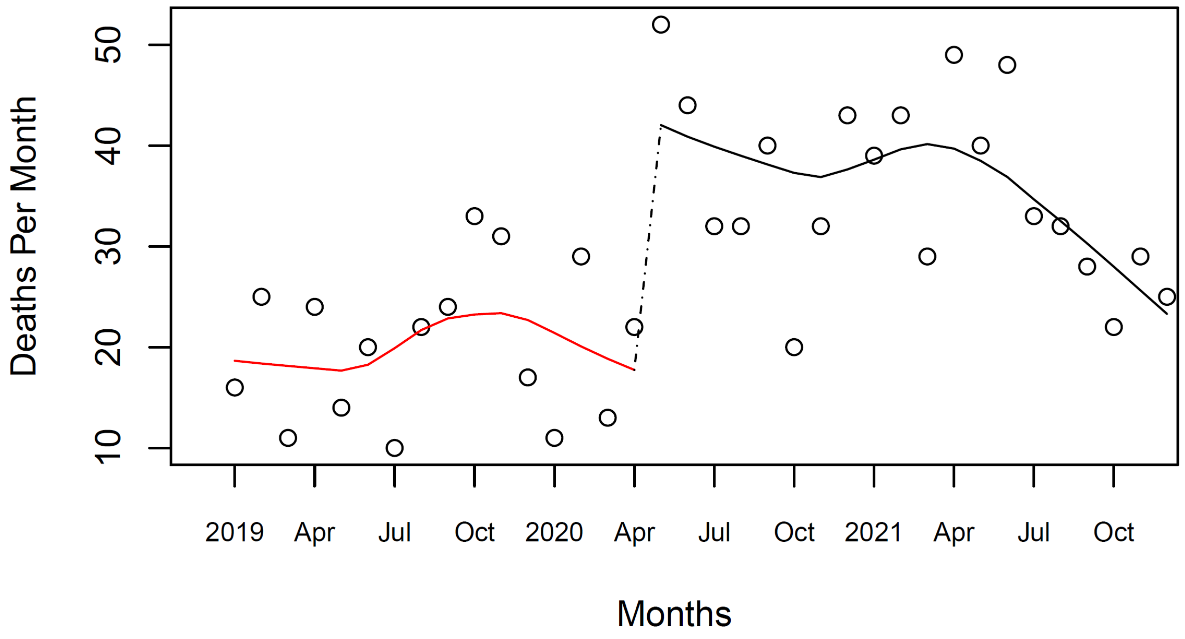

Supplement Figure 2. Monthly Overdose Deaths Caused by Heroin in Pinellas County Before, At the Beginning, and During the Pandemic, 2019-2021

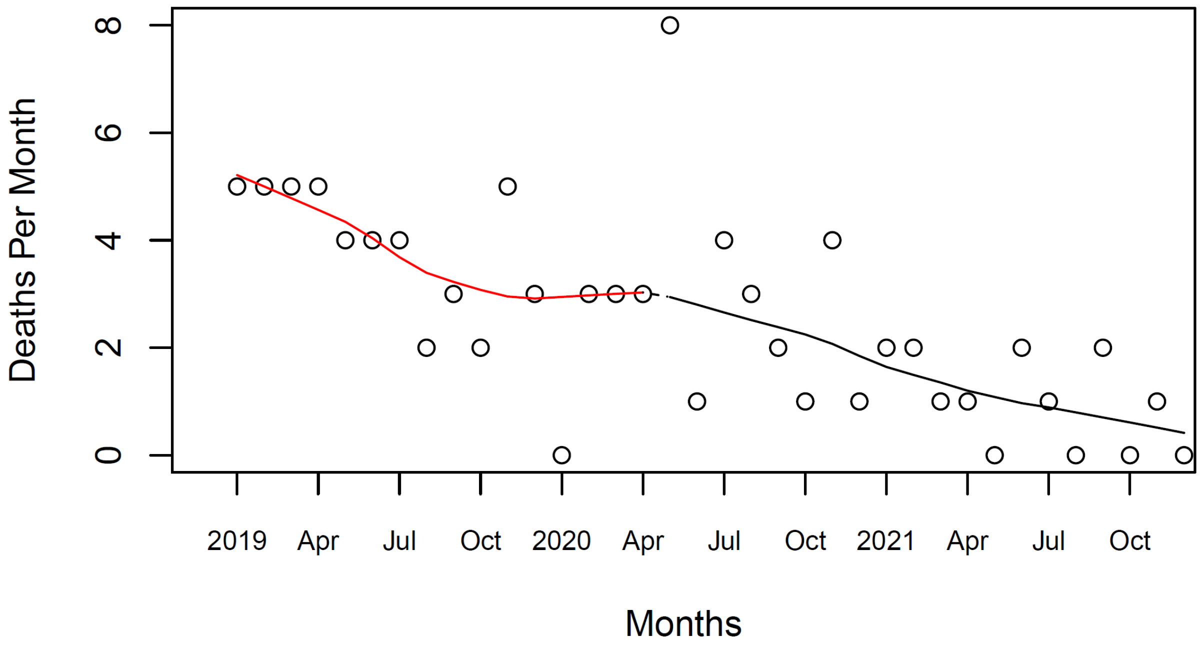

Supplement Figure 3. Monthly Overdose Deaths Caused by Amphetamines in Pinellas County Before, At the Beginning, and During the Pandemic, 2019-2021

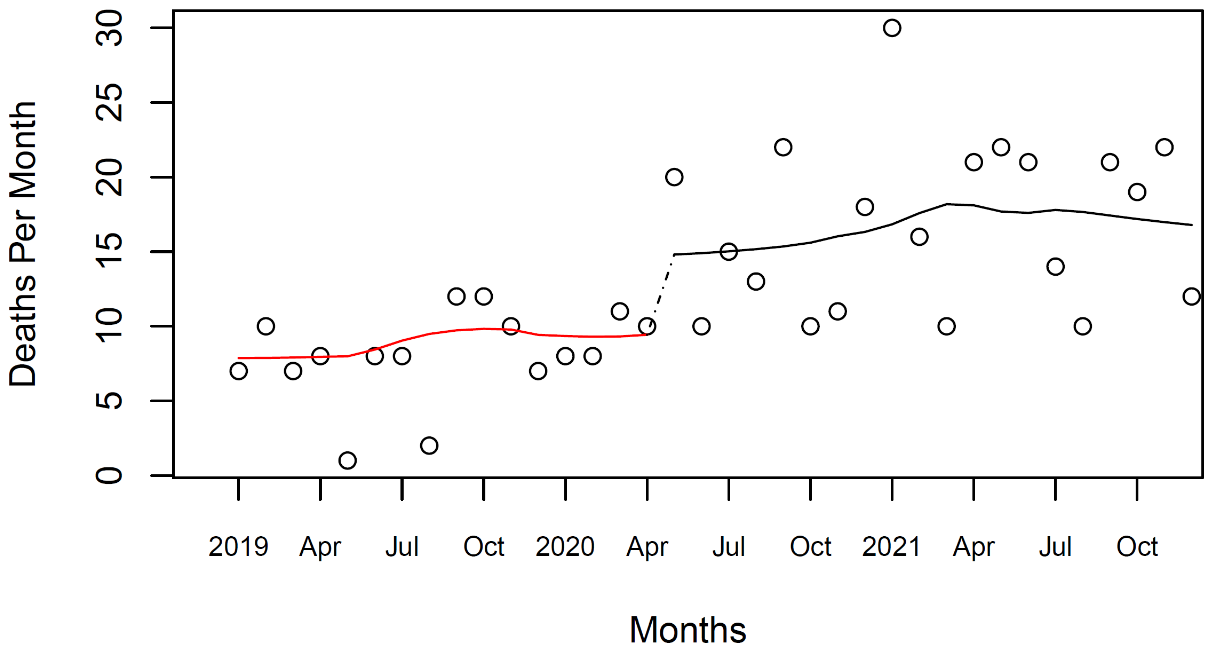

Supplement Figure 4. Monthly Overdose Deaths Caused by Cocaine in Pinellas County Before, At the Beginning, and During the Pandemic, 2019-2021

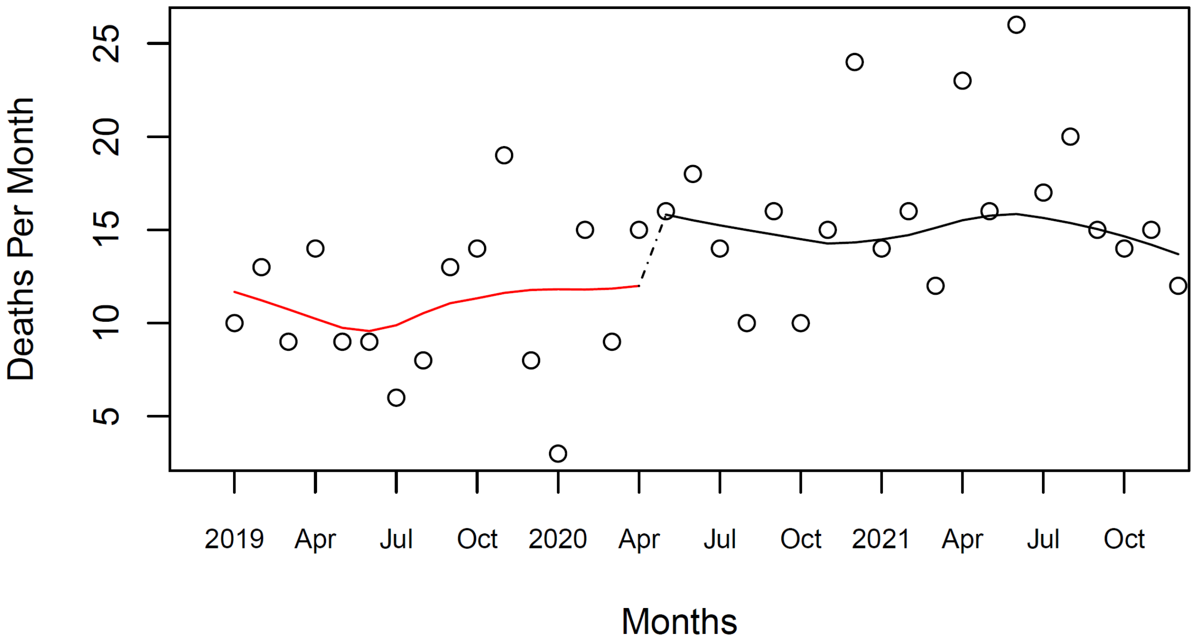

Supplement: Supplementary file 1 [file Image_1.pdf]
